# Efficacy of Externally Paced Training on Pain in Tendinopathy: A Systematic Review and Meta Analysis

**DOI:** 10.7759/cureus.39994

**Published:** 2023-06-05

**Authors:** Dylan Carmody, Alyssa Conanan, Daniel Moeller, Sarah Khoblall, Christopher Keating

**Affiliations:** 1 Physical Therapy, Thomas Jefferson University, Philadelphia, USA

**Keywords:** sports injury rehabilitation, mechanical pain, progressive resisted exercise, orthopaedics and sports physical therapy, tendinopathy pain

## Abstract

Tendinopathy is a common condition with treatments focused on local tissue adaptations. Externally paced loading programs are designed to cue (visually, auditorily, or temporally) a person as to when to perform an exercise repetition during a set of repetitions. Externally paced loading programs propose central and peripheral changes with tendinopathy but conclusions regarding their efficacy on pain outcomes remain limited. Our review seeks to explore the efficacy of externally paced loading as a method to reduce self-reported pain in tendinopathic conditions. An electronic database search was conducted of PubMed, SPORTDiscus, Scopus, and CINAHL databases. A total of 2,104 studies were identified after a preliminary search; four reviewers narrowed the selection to seven articles based on inclusion and exclusion criteria. Articles selected for review (patellar = three, Achilles = two, rotator cuff = one, and lateral elbow tendinopathy = one) were randomized control trials assessing the externally paced loading programs’ efficacy on tendon pain compared to the control; all were included in the meta-analysis. This review identified no superiority in externally paced loading compared to alternative treatment. There were potential population differences between non-athletic and athletic populations as identified with subgroup analyses. Current activity levels, region of tendinopathy, and chronicity of symptoms may explain the variability of findings. There is little clinically significant evidence to support the use of externally paced loading programs for reducing tendon pain over standard clinical care based on a low level of certainty which is based on the Grading of Recommendations, Assessment, Development, and Evaluations (GRADE) of articles included in the review. Clinicians should interpret outcomes between athletic and non-athletic participants with caution as further high-quality studies are required to confirm specific clinical outcomes in these populations.

## Introduction and background

Tendinopathies are common, widespread tendon injuries that affect the entire population, and are seen with a greater prevalence in athletes and those with high occupational demand [1]. While it is unlikely for a single model to encompass the entirety of tendon pathoetiology, tendinopathies are characterized by phases of cellular response to mechanical stimuli [2]. In its original proposition [3], the tendon continuum model proposed a staged presentation of tendinopathy intended to influence the delineation of interventions within each proposed stage. This model serves as a framework for tendon pain and function.

Tendon pain seems to only be moderately correlated to tendon pathology as tendons that present with histopathological changes can be without symptoms, and tendons that present with normal morphology can be painful [4,5]. Treatments for tendinopathy should be targeted to improve patient symptoms and irritability, rather than change morphology [6]. Recurrence rates and duration of symptoms for tendinopathy are highly variable due to the heterogeneous population but are high for athletes with patellar and Achilles tendinopathy which can lead to pain and persistent impairments in athletic performance [7-9].

While activity seems to play a major role in the development of tendinopathy, there are various intrinsic and extrinsic variables that increase the likelihood of developing a tendon-related injury. Range of motion [10,11], age [12], and biological sex pose as intrinsic risk factors in developing tendinopathy [13,14], whereas environmental influences, such as training on a concrete floor [5], and medication use have also been seen to increase tendinopathy risk [15-17].

There is currently no single effective treatment strategy for the management of tendinopathy [18]. While the current state of evidence points toward loading protocols targeting the symptomatic structures, there is no clear consensus on which protocol is best. Evidence supporting the utilization of eccentric exercise permeates the tendinopathy literature [19-21], however, other evidence suggests that eccentric exercise may have reduced effectiveness for in-season and elite athletes [22,23]. Variables such as muscle recruitment rate, muscle type, motor planning, and motor control may relate to the recurrence rate of tendinopathic conditions. External pacing is defined as an external cue (visual, auditory, or temporal) to address the central motor impairments (recruitment rate, motor planning, or motor control) seen in those with tendinopathy while implementing the known local benefits of tendon loading programs. Heavy slow resistance training (HSR) is a recent, temporally sequenced treatment strategy that involves an isotonic, concentric-eccentric muscle contraction which has similar effectiveness to eccentric exercise [20], and provides analgesia for athletes in a competitive season [24,25]. Even more recently, tendon neuroplastic training (TNT) has emerged as a potential treatment option utilizing either auditory or visual cues. Proposing changes in corticospinal excitability and cortical inhibition due to external pacing factors which alter one’s motor program for a given task, TNT appears to be a viable option that addresses tendinopathy at the peripheral site of injury, as well as the top-down neurological processing of activity with a painful tendon [18]. Isometric exercises are not included in the external pacing definition as an individual's motor plan is likely unchanged when performing the exercise.

While damage or inflammation-induced nociception is likely a factor in tendinopathy-related pain conditions, it cannot be the only considered reason for such pain experiences [26,27]. Evidence is emerging for centrally-mediated mechanisms to play a role in pain-processing for tendinopathy and may be a point of intervention for those experiencing persistent symptoms [28,29]. Loading protocols and interventions from the literature have consistently addressed the local structural changes of a tendon in an attempt to alter symptoms of tendinopathy, however, the poor association between pain and structure points to the need for interventions to address the multidimensional nature of pain.

To our knowledge, this is the first systematic literature review to assess the efficacy of externally paced loading programs on tendon pain. The use of external pacing is limited in the literature and within the use of external pacing, the parameters of the loading programs and types of cueing provide no clear consensus. Based on the level of current evidence in tendinopathy treatment and known gaps, the identification of the efficacy of external pacing on tendinopathy could provide clinically meaningful guidance in the management of tendinopathy. 

The objective of this review is to answer the research question - in adults diagnosed with tendinopathy, is externally paced loading an efficacious treatment technique to decrease self-reported pain outcomes? This systematic review aims to explore the efficacy of externally paced, loaded exercise as a method to reduce self-reported pain in tendinopathic conditions.

## Review

Materials and methods

This systematic literature review was performed according to the protocol guidelines described in the Preferred Reporting Items for Systematic Reviews and Meta-Analyses (PRISMA) statement [30]. Selected trials were assessed for risk of bias using the Consolidated Standards of Reporting Trials Statement [31].

**Figure 1:**
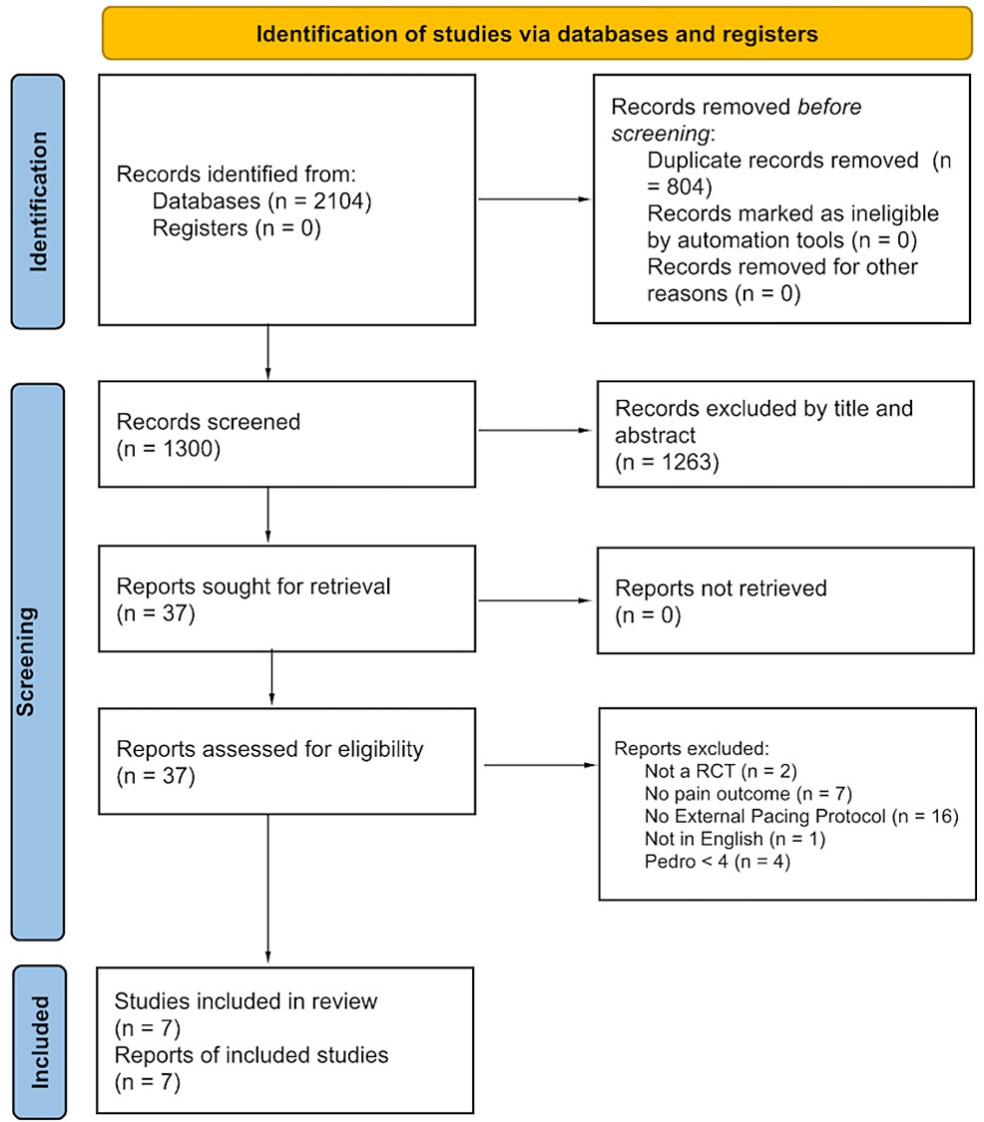
Preferred Reporting Items for Systematic Reviews and Meta-Analyses (PRISMA) flow chart

Studies that included tendinopathy populations, reported pain outcomes, and utilized loading or resistance training were included in this review. Randomized Controlled Trials (RCT) that investigated externally paced loading protocols (timed eccentric, HSR, or TNT) with a primary outcome of pain were targeted for review. Studies were not reviewed if they were written in a language other than English, published outside of 10 years from the review date, did not include pain as a primary outcome measurement, included pediatrics (age < 18), did not report an externally paced loading protocol, PEDro score < 4/10, or an RCT study design (Figure 1). An electronic database search was conducted of PubMed, SPORTDiscus, Scopus, and CINAHL databases on September 23, 2021. Articles published from January 2011 to September 2021 were included in the search. A flowchart of specific search terms for each database can be found in Table 1. 

**Table 1:** Search terms

PubMed	Scopus	CINAHL	SPORTDiscus
("Tendinopathy"[Mesh] OR Tendinopathy OR Tendinosis OR Tendinitis OR Paratenonitis OR peritendinitis OR tendi* OR teno* or tendo*)	((Tendinopathy OR Tendinosis OR Tendinitis OR “Tendon Pain” OR “Tendon Related Pain” OR Paratenonitis OR peritendinitis OR tendi* OR teno* or tendo*)	(MH “Tendinopathy+”) OR Tendinopathy OR Tendinosis OR Tendinitis OR Paratenonitis OR peritendinitis OR tendi* OR teno* or tendo)	Tendinopathy
AND			
(tendon neuroplastic training OR Heavy Slow OR Heavy-Slow OR Resistance OR Training OR isotonic OR Neuroplastic training OR external timing OR Cortical Reorganization OR paced OR Metronome OR Neuronal plasticity OR load* OR neuroplasticity OR resistance training)	(training OR “tendon neuroplastic training” OR “Neuroplastic training” OR “neuronal plasticity” OR Metronome OR isotonic OR “heavy slow” OR “heavy-slow” OR Paced OR Timing OR “Resistance Training”)	(tendon neuroplastic training OR Heavy Slow OR Heavy-Slow OR Resistance OR Training OR isotonic OR Neuroplastic training OR external timing OR Cortical Reorganization OR paced OR Metronome OR (MM “Neuronal Plasticity”) OR Neuronal plasticity OR load* OR neuroplasticity OR resistance training)	Tendon neuroplastic training OR Heavy-Slow OR Resistance OR Training OR cortical reorganization OR isotonic OR neuroplastic training OR external timing OR paced OR metronome OR neuronal plasticity
AND			
(Pain OR "Pain Measurement"[Mesh] OR self-reported pain Or "Pain"[Mesh] OR "Self Report"[Mesh] OR "Treatment Outcome"[Mesh]) AND (Randomised Control* OR Controlled)	(“Musculoskeletal Pain” OR pain OR “Pain Measurement” OR “self-reported pain”))	(MM “Pain+”) OR Pain OR self-reported pain OR (“MM Self Report+”) OR (“MM Treatment Outcomes+”) AND (Randomised Control* OR Controlled Clinical Trial OR Randomly)	Pain OR Self report OR Treatment Outcome

Studies with a publication date greater than 10 years were excluded as the concept of external pacing was originally introduced within the past five years [18]. In prior literature, internal and external cueing were not clearly defined, therefore narrowing the search strategy allows the authors to assess the available research of the current topic within a reasonable and representative timeframe. 

Four reviewers screened the titles of all articles curated from the search strategy for relevance to the search topic in Sciwheel (www.sciwheel.com.lg (operated by SAGE Publications Limited, a company registered in England and Wales with company number 01017514, with registered offices at 1 Oliver's Yard, 55 City Road, London, EC1Y 1SP)). Reviewers then screened abstracts for inclusion and exclusion criteria; if relevant information was not presented in the abstract, reviewers screened the methods of a potential article. Full text review was only performed once articles met the aforementioned inclusion and exclusion criteria. Final articles were reviewed and scored via the Physiotherapy Education Database score for Randomized Controlled Trials (PEDro) scale; papers that failed to score 4/10 on the PEDro scale were excluded. Five reviewers met and discussed PEDro items and scores until a group consensus was determined. Independent article review was performed to decrease the risk of bias prior to group discussion. Seven studies were included in the review. Participant demographics, intervention protocols, group sizes, pain measures, and pain rating scores are listed in Table 2.

**Table 2:** Characteristics of studies Abbreviations: ECC= eccentric, HSR= heavy slow resistance, CG= control group, VIB= vibration, WAS= wait and see, ISO-T= isotonic, ISO-M= isometric, IFR= inertial flywheel resistance,VAS= Visual Analogue Scale, VISA-P= Victorian Institute of Sports Assessment-Patellar Tendon, NPRS= Numeric Pain Rating Scale

Study	Number of participants in study	Number Female	Population	Patient Diagnosis	Groups (size)	Duration of Intervention (weeks)	Frequency (days/week)	Length of Follow-Up (weeks)	Primary Outcomes
Dejaco et al. (2017) [19]	36	17	Non-Athletic	Rotator Cuff Tendinopathy	ECC + stretching (20) CG + stretching (16)	12 weeks	7 days/week	26 weeks	VAS
van Ark et al. (2016) [24]	29	2	Athletic	Patellar Tendinopathy	ISO-T (8) ISO-M (11)	4 weeks	4x/week	4 weeks	NPRS VISA-P
Rio et al. (2017) [25]	29	Not reported	Athletic	Bilateral Patellar Tendinopathy	ISO-T (10) ISO-M (10)	4 weeks	3x/week	4 weeks	VISA-P
Kedia et al. (2014) [32]	36	26	Non-Athletic	Insertional Achilles Tendinopathy	ECC (16) CG (20)	12 weeks	2x day 7 days/week	12 weeks	VAS
Ruffino et al. (2021) [33]	42	1	Athletic	Unilateral Patellar Tendinopathy	HSR (21) IFR (20)	12 weeks	3x/week	12 weeks	VISA-P
Horstmann et al. (2013) [34]	58	36	Non-Athletic	Chronic Achilles Tendinopathy	VIB (22) ECC (18) WAS (14)	12 weeks	3x/week	12 weeks	VAS
Wen et al. (2011) [35]	28	13	Non-Athletic	Lateral Epicondylosis	ECC (14) CG (14)	12 weeks	2x/week for 2 weeks, 1x/week for 12 weeks	20 weeks	VAS

Results

**Figure 2:**
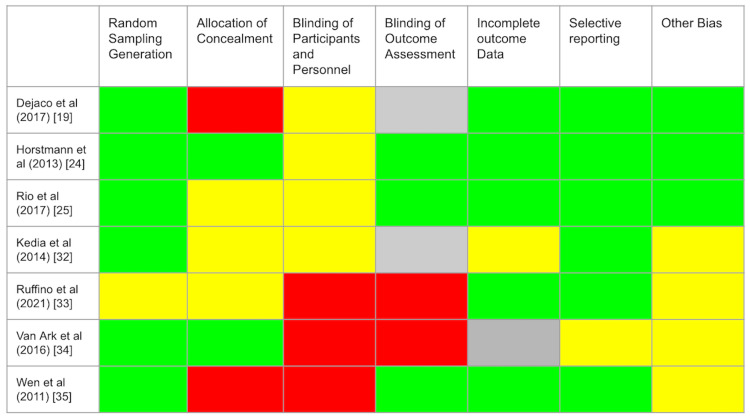
Risk of bias assessment (RoB 2) tool Green: low risk of bias, Yellow: medium risk of bias, Red: high risk of bias, Grey: undetermined.

Two authors performed a risk of bias assessment (RoB 2) tool using the latest framework provided by Cochrane of the seven RCTs in this review [36]. Refer to Figure 2 for further details regarding this assessment. 

Relevant information from the screened articles was extracted by three reviewers; data included baseline and post-intervention participant characteristics, loading intervention protocols, pain outcome measures (Visual Analogue Scale (VAS), Victorian Institute of Sport Assessment (VISA), and Numeric Pain Rating Scale), and threats to internal and external validity within the study.

Participant reports of pain were extracted from the list of accepted articles via multiple methods to standardize group means. Due to the lack of standardization for tendinopathy-related pain assessment, group data of pain reports were extrapolated to a 0-100 VAS. The VISA scoring method was transferred to VAS without manipulation, as each representative tool (VISA-P and VISA-A) scores pain and function on a 0-100 scale. Studies assessing pain reports via 0-10 assessment scale were extrapolated to 0-100 VAS [24,32].

Standardized means and standard deviations (SD) of pre and post-intervention group data were collected to determine the effect size of pain change between groups. Reviewers calculated the SD of pain report means from confidence intervals when SD were not reported. Effect size means and SD of pain data were utilized to calculate standardized differences between means (Cohen’s d) via SPSS, version 15 (IBM Corp., Armonk, NY), for analysis.

**Figure 3:**
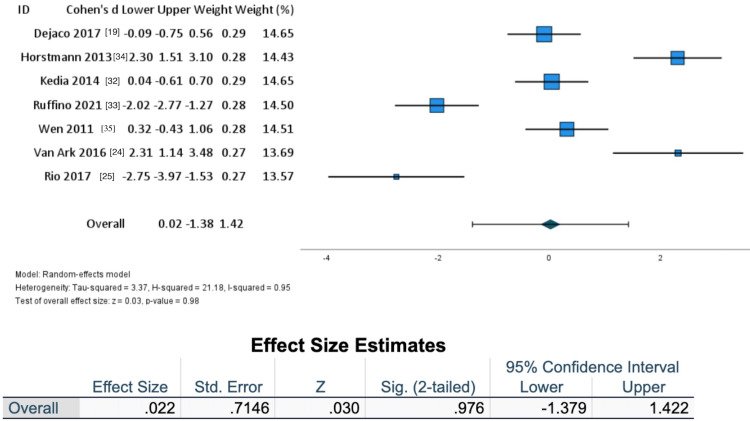
Forest plot

**Figure 4:**
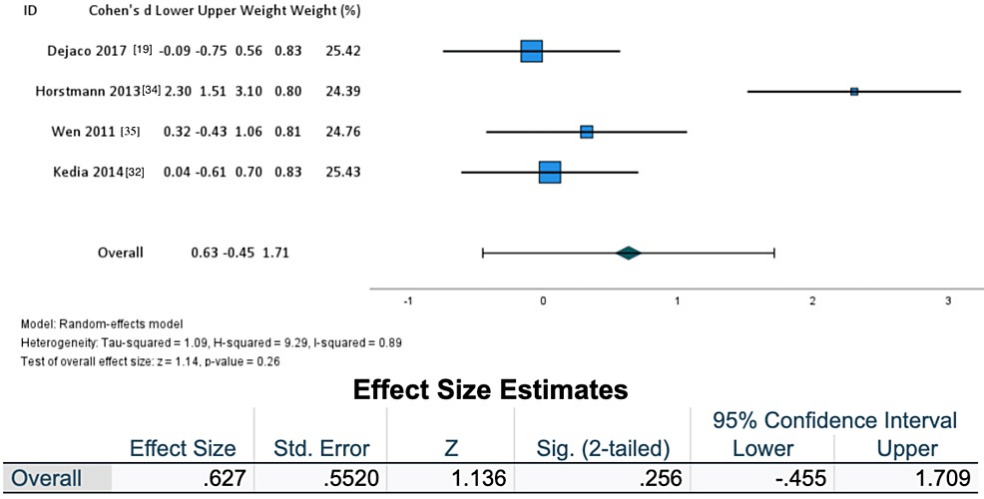
Non-athletic population forest plot

**Figure 5:**
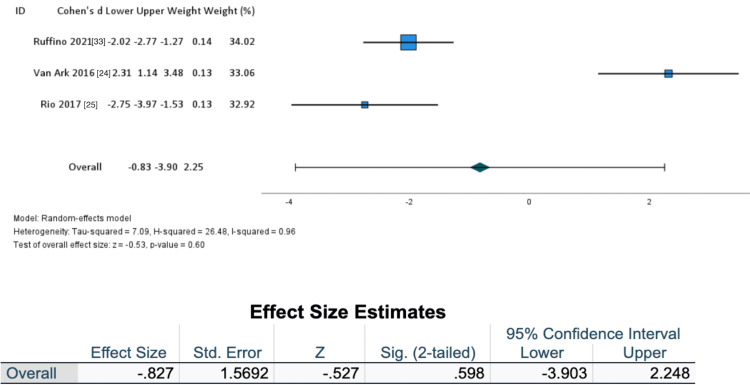
Athletic population forest plot

Researchers collected changes in group means and SDs to formulate a forest plot (Figure 3). Means and SD of effect sizes within groups were included from each study. A subgroup analysis was performed to address treatment sensitivity in the non-athletic population compared to an athletic population (Figures 4-5).

All reviewed articles were included in a meta-analysis as the involved studies had sufficient homogeneity in participant characteristics, exercise protocols, therapy treatments, and pain outcome questionnaires. Interpretation of effect sizes were conducted where a Cohen’s d value below 0.2 was considered negligible, between 0.2-0.5 was considered a small effect, 0.5-0.8 was interpreted as a medium effect, and anything over 0.8 was large. 

A forest plot including all articles is shown in Figure 3, where negative values favor the controlled, self-paced exercise groups and positive values favor an externally paced loading protocol. A small and insignificant difference was observed with an overall effect size of 0.022 (Z = 0.030, SE = 0.7146, I2 = 0.95) favoring an externally paced exercise protocol. 

After further assessment, a subpopulation analysis was performed to assess the utility of externally paced exercise for tendon-related pain in the non-athletic population (as seen in Figure 4) and an athletic population group (Figure 5). A larger overall effect size was seen in the non-athletic population (Cohen’s d = 0.627, Z = 1.136, SE = 0.5520, I2 = 0.89) when compared to an athletic population (Cohen’s d = -0.827, Z = 0.527, SE = 1.5692, I2 = 0.96).

Discussion

This study examined seven high-quality RCTs in which a definitive comparison group was not specified. The comparison groups included biophysical agents, stretching, conventional physical therapy treatment, and comparable loaded exercise strategies. The majority of studies reviewed were unable to find significant differences between the randomized groups, making definitive conclusions about externally paced loading programs unclear. Externally paced loading programs appear to produce similar pain outcomes for those with tendinopathy when compared to the standard of rehabilitative care.

Pathological changes present themselves within the tendons of athletes and non-athletes alike, however, rarely do clinical trials compare tendon loading programs between such populations [37]. While the underlying mechanisms by which tendinopathy develops are consistent, the mechanisms of tendon healing, reconditioning, and pain reduction remain undiscovered for specific subpopulations. A subgroup analysis of the involved articles suggests that externally paced resistance exercise in a non-athletic population may be beneficial (Figure 4). 

Several underlying factors may explain the difference between the studies featuring athletic and non-athletic participants. Athletes may respond less favorably to externally paced exercise as they may exhibit superior, innate coordinative strategies to perform exercises, reducing the need for external pacing to improve motor-skill acquisition. Similarly, those without an athletic background have less experience with exercise and training, which may increase their sensitivity to a stimulus aimed to alter muscular recruitment and motor programming. An alternative factor may be that such findings are the result of a type I error due to an over-analysis of data. Regardless of the findings, the authors call for further research to assess adaptations of externally paced exercise between athletic and non-athletic populations for more conclusive evidence to support or refute these findings. In addition, the lack of research on the utilization of externally paced loading programs in labor intensive occupations, despite the high prevalence of injury in this population, is a gap in the literature that needs further research. 

Pain is a multidimensional, biopsychosocial experience that is challenging to measure using outcome measures, scales, and graphs. The inconsistencies with an individual’s experience with pain are also reflective of the inconsistencies in researchers’ and clinicians’ preferred tool of pain measurement. Such claims have been reinforced with recent evidence suggesting that clinical trials continue to remain inconsistent in their utilization of accepted outcome measures [36]. Our current review examines seven articles that measured pain with three separate measurement tools (VISA-P, VAS, NPRS). Such inconsistencies in measurement, as well as the inherent variability in the construct of pain, present as threats to the internal validity of the review.

Tendon healing time is highly variable and can take anywhere between six and 12 months to return to pre-injury capacity. Similarly, structural deformity and altered muscle recruitment patterns may remain years after pain has been absolved, making tendon healing a difficult target to define [38]. The average length of intervention for the included papers was approximately 12 weeks, a remarkably short duration for the assumption of tendon healing to occur. Similarly, the length of follow-up in the included studies ranged from four weeks to 26 weeks, failing to meet an accepted time frame for tendon healing. 

The rate of pain measurement was highly variable across the involved studies. Three papers assessed pain twice; at baseline and follow up [24,25,34], while the remaining four articles captured pain outcomes at numerous times throughout the trials [19,32,33,35]. Threats to internal validity may ensue in trials that use the same outcome measure frequently due to participant familiarity and memory of prior scores through testing effects. Standardization of pain assessment times for future RCTs may lead to reducing this threat.

Chronicity of tendinopathy within the sample populations was only assessed by two of the seven reviewed articles [34,38], and was only accounted for on the basis of time. Staging of tendinopathy along the continuum model may explain the heterogeneity of responses in the studied populations and meta-analysis (I^2^ = .95). While current evidence denies the necessity for medical imaging of individuals with tendinopathy, we propose that future trials look towards creating more specific diagnostic criteria to categorize tendinopathic changes into the four stages outlined by Cook and Purdam [3]. 

Tendinopathy has the potential to appear in any tendon experiencing an acute increase in workload across the body creating variability in the regions that are affected by the condition. Out of the seven reviewed articles, only two focused on upper extremity presentations of tendinopathy [19,38]. Due to its lack of representation, the evidence presented in our review may not accurately represent the effect of externally paced exercise on individuals with upper extremity tendinopathy. Similarly, evidence is lacking comparisons of effective loading strategies between upper and lower extremity tendinopathy. 

Implementing the Grading of Recommendations, Assessment, Development, and Evaluations (GRADE) tool [39], the authors present the findings from this study with low certainty that the estimated effect of externally paced training protocols on improving subjective pain outcomes represents a true effect within the populations studied. Limitations arising from the imprecision of data based on the limited number of included studies, inconsistency of findings due to overlapping of confidence intervals, and degree of statistical heterogeneity exist. High likelihood of bias among the included studies currently impedes the authors' confidence that the data presented are representative of the populations assessed. These recommendations and grading decisions are subjective interpretations of the authors’ perception of the reviewed literature. The authors suggest further efforts be made to accurately define tendinopathy sub-groups, and standardize pain assessments to gather more representative and precise data of these populations.

This systematic review had several limitations. The study search was constrained to articles written in English and within 10 years of the search date. The authors recognize this could lead to cultural bias, but as highlighted in Figure 1, only one article was removed for being published in a language other than English. The reviewed articles fail to capture a high occupational demand population with a known high incidence of tendinopathy. Several reviewed articles’ sample population presented with male sex/gender bias. In addition, the authors recognize that the small sample size is a limitation. 

## Conclusions

This systematic literature review and meta-analysis analyzed the evidence of externally paced loading protocols on various pain outcomes. Although limited and with low certainty, the evidence examined fails to find a superior benefit in the utilization of externally paced loading in reducing pain over conventional therapies. While loading protocols utilizing external pacing were unable to demonstrate superior pain outcomes compared to traditional programs, they show a possible benefit in non-athletic populations. 
